# Retrovirus insertion site analysis of LGL leukemia patient genomes

**DOI:** 10.1186/s12920-019-0549-9

**Published:** 2019-06-17

**Authors:** Weiling Li, Lei Yang, Robert S. Harris, Lin Lin, Thomas L. Olson, Cait E. Hamele, David J. Feith, Thomas P. Loughran, Mary Poss

**Affiliations:** 1 The School of Electrical Engineering and Computer Science, The Pennsylvania State University, University Park, PA 16802 USA; 2 Department of Biology, The Pennsylvania State University, University Park, PA 16802 USA; 3 Department of Veterinary and Biomedical Sciences, The Pennsylvania State University, University Park, PA 16802 USA; 4 Department of Statistics, The Pennsylvania State University, University Park, PA 16802 USA; 5 University of Virginia Cancer Center and Department of Medicine, Division of Hematology & Oncology, University of Virginia, Charlottesville, Virginia 22908 USA

**Keywords:** Large granular lymphocyte leukemia, Retrovirus, HERV-K, Genomic insertion, Visualization tool

## Abstract

**Background:**

Large granular lymphocyte (LGL) leukemia is an uncommon cancer characterized by sustained clonal proliferation of LGL cells. Antibodies reactive to retroviruses have been documented in the serum of patients with LGL leukemia. Culture or molecular approaches have to date not been successful in identifying a retrovirus.

**Methods:**

Because a retrovirus must integrate into the genome of an infected cell, we focused our efforts on detecting a novel retrovirus integration site in the clonally expanded LGL cells. We present a new computational tool that uses long-insert mate pair sequence data to search the genome of LGL leukemia cells for retrovirus integration sites. We also utilize recently published methods to interrogate the status of polymorphic human endogenous retrovirus type K (HERV-K) provirus in patient genomes.

**Results:**

Our data show that there are no new retrovirus insertions in LGL genomes of LGL leukemia patients. However, our insertion call tool did detect four HERV-K provirus integration sites that are polymorphic in the human population but absent from the human reference genome, hg19. To determine if the prevalence of these or other polymorphic proviral HERV-Ks differed between LGL leukemia patients and the general population, we used a recently developed tool that reports sites in the human genome occupied by a known proviral HERV-K. We report that there are significant differences in the number of polymorphic HERV-Ks in the genomes of LGL leukemia patients of European origin compared to individuals with European ancestry in the 1000 genomes (KGP) data.

**Conclusions:**

Our study confirms that the clonal expansion of LGL cells in LGL leukemia is not driven by the integration of a new infectious or endogenous retrovirus, although we do not rule out that these cells are responding to retroviral antigens produced in other cell types. However, our computational analyses revealed that the genomes of LGL leukemia patients carry a higher burden of polymorphic HERV-K proviruses compare to individuals from KGP of European ancestry. Our research emphasizes the merits of comprehensive genomic assessment of HERV-K in cancer samples and suggests that further analyses to determine contributions of HERV-K to LGL leukemia are warranted.

**Electronic supplementary material:**

The online version of this article (10.1186/s12920-019-0549-9) contains supplementary material, which is available to authorized users.

## Background

Large granular lymphocyte (LGL) leukemia is a rare, chronic, proliferative disorder of cytotoxic T cells (approximately 85% of cases) and NK cells [1]. Diagnosis of this leukemia is based on a sustained elevation of a clonally expanded T or NK cell population. LGL leukemia is reported most frequently in patients from North America and Europe and up to half of patients also have an autoimmune disorder, most frequently rheumatoid arthritis [2]. A small subset of patients show clonal proliferation of CD4+ cells, which has been associated with Cytomegalovirus infection [3]. An aggressive form of LGL leukemia involving natural killer cells, (NK leukemia) is most common in East Asians and has been linked with Epstein-Barr virus infection [4]. Aspirates demonstrate close approximation of LGL and antigen presenting cells [5], emphasizing that prolonged presentation of an unknown antigen could be a common underlying feature of the various forms of LGL leukemia. At present, it is unknown if an infectious agent is responsible for chronic antigen stimulation of LGL in some or all patients, although non-malignant proliferation of LGL does occur in chronic viral infections [6].

Serum antibodies from LGL leukemia patients recognize an antigen with homology to a protein encoded by human T-lymphotropic virus (HTLV) [7, 8], providing a potential link of this disease with retroviruses. The oncogenic potential of retroviruses is well established in mammals and birds, which can develop cancers of hematopoietic cells following retroviral infection; reviewed in [9]. Although there are numerous mechanisms for retrovirus-induced oncogenesis, dysregulation of key cell cycle control genes during retroviral integration and transduction of cellular oncogenes are particularly well documented [9–11]. While many cancers in animals have a retrovirus etiology, HTLVs are the only retrovirus group definitively linked to cancer in humans. In this case, virus-encoded accessory proteins are necessary for cell transformation [12–14]. Despite a high prevalence of HTLV-1 antigen responders among LGL leukemia patients, intensive molecular and culture-based approaches have failed to detect HTLV, or any known retrovirus, in LGL leukemia patients. However, such methods could fail to identify a defective retrovirus, which is relevant because many animal oncogenic retroviruses are replication-defective and can have unusual genome sequence [15, 16]. Because LGL leukemia involves a clonal expansion of LGL cells, we reasoned that if a retrovirus initiated the malignancy, we should be able to detect the integrated virus in LGL genomes even if it couldn’t be recovered in culture. The goal of this study was to interrogate LGL genomes of LGL leukemia patients for the presence of an unknown retroviral insertion to determine if the clonal expansion of LGL cells is preceded by the integration of a novel or known retrovirus.

## Methods

### Patient samples

LGL leukemia patients met the clinical criteria of T-LGL leukemia with increased numbers of CD3^+^, CD8^+^/CD57^+^ T lymphocytes or CD3^−^, CD16^+^/CD56^+^ NK cells in the peripheral blood [17]. LGL leukemia patient blood samples were obtained and informed consents signed for sample collection according to the Declaration of Helsinki using a protocol approved by the Institutional Review Board of the University of Virginia. Blood was subjected to Ficoll-Hypaque (Sigma Aldrich) gradient centrifugation for peripheral blood mononuclear cell (PBMC) isolation.

### Whole genome sequencing (WGS)

Long insert mate pair libraries of 11 LGL patients (S1-S11) were prepared and sequenced at the Duke Center for Genomic and Computational Biology using the Illumina Nextera MP kit. Sequencing was performed on a HiSeq and read length was 125 bp. Paired-end sequencing of 48 LGL patients (including patients S1-S8) was conducted by Illumina. Patients S9, S10, and S11 were paired-end sequenced at Penn State by Dr. Stephan Schuster.

### PCR assessment of four non-reference polymorphic HERV-K

A 25 μl PCR reaction was performed using human adult normal peripheral blood leukocyte genomic DNA purchased from Biochain (Cat. #D8234148–1, Lot #B511221) and the following primer pairs:

- Chr1_5HK For (5’CATAGCAAATCCCAGTGTAGACATC3’) and
- Chr1_5HK Rev. (5’CTGGGAGCATTTCTGGACATC3’);
- Chr1_PREINT For (5’CACCGCACCTGGCAAGTTTACA3’) and
- Chr1_PREINT Rev. (5’ATTTGGGGTCCTCATGAAGCAGAA3’);
- Chr19_3HK5 For (5’TACCCCAAGACCAAAAATAATAAG3’) and
- Chr19_3HK6 Rev. (5’CTGATAGTGGCAAGATGGATGTA3’);
- C19PRE3 For (5’GAACAGGAGCATGCTCATAGTGTGT3’) and
- C19PRE4 Rev. (5’GGTCTCGAACTCCTAACCTCCTG3’);
- Chr12PREINT For (5’TACTGGGAATAAGATGATGATGGT3’) and
- Chr12PREINT Rev. (5’TTTGTTAAGTGCTCGGAAGGT3’);
- Chr10HK1 For (5’ACGCGTGGGTATATCGGTTTATTTCT3’) and
- Chr10HK2 Rev. (5’GGCTGGTTCTTTATTATTTATGGCTGGT3’);
- Chr12HK5_1 For (5’GCAGTTCCACCTTCCCGACAGC3’) and
- Chr12HK5_2 Rev. (5’ACACAGGACCAAAAGAACGAGTAATC3’).

Each 25 μl reaction contained 12.5 μl 2x MyTaq HS Red Mix, 2 μl 5 μM forward primer, 2 μl 5 μM reverse primer, 3.5 μl nuclease-free water and 20 ng of the pre-dispensed genomic DNA. The reactions were cycled using a Bio-Rad C1000 Touch 96-deep well cycler. The cycled reaction and HyperLadder 1 kb DNA ladder were electrophoresed on a 1% agarose gel with 1x TAE for 60 min at 80 V. The gel was visualized using 10,000x Sybr Safe DNA Gel Stain and images were captured using the Bio-Rad ChemiDoc and Image Lab software.

### Insertion call pipeline

**Fig. 1:**
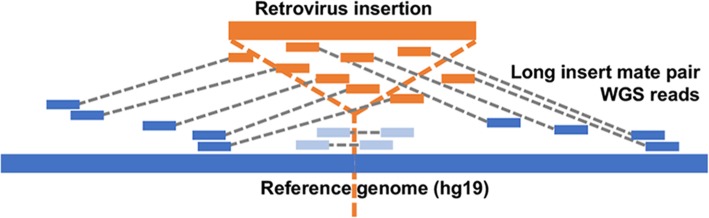
Utilizing long insert mate pair reads to localize retrovirus integrations. Reference human genome is shown as a blue line with the location of a novel inserted retrovirus in a patient sequence, in orange, indicated by a dotted vertical orange line. Long insert mate pair reads are linked by gray dotted lines, with the read derived from the new retrovirus, which will not map, shown in orange, it’s mate that maps to the human reference genome shown in blue. Depending on the length of the retrovirus, which typically is 6–10 kbp, some mate pairs may span the entire inserted virus and hence both mate pairs will originate from the host (light blue), resulting in mate pairs that map at a distance shorter than the expected insert distribution of 5–12 kbp. A retrovirus insertion site is suggested by a combination of several features of mate pair mapping including short insert intervals and discordant or broken mate pairs. The insert length and depth of mapped reads are key signals in our retrovirus insertion pipeline (see Additional file 1: Supplementary Methods; Figure S1). The unmapped reads (orange in the figure) from discordant mate pairs at each called insertion site are assembled and used to determine the sequence of a candidate retrovirus

A detailed description of the insertion call pipeline is given in Additional file 1: Supplementary Methods. Briefly, sites containing a retroviral insertion result in aberrant mapping of long insert mate pairs to the reference genome. We developed a series of signal tracks to detect insertions that include shorter than expected insert length of mate pairs, distant or inter-chromosomal mapping of mate pairs, orphan mate pairs where only one read of the pair can be mapped, and partially mapped reads (Fig. 1). Parameters of the caller were tuned using a simulation to detect 5–12 kbp insertions of at least 55% prevalence in a sample (see Additional file 1: Supplementary Methods). Five tracks were integrated to identify candidate insertion events. The sequence content of each candidate insertion was investigated by gathering the long insert read pairs that map near the insertion, assembling them, and querying the resultant contigs, which contain the sequence of the inserted element, against the NCBI nt database using BLAST. BLAST results were searched for infectious or endogenous retrovirus hits based on taxonomy identification (Additional file 1: Figure S3). Endogenous retrovirus hits that had host flanking regions were confirmed to be from the candidate insertion site by mapping to the reference genome. A second method independent of the insertion call pipeline was used to confirm detected polymorphic HERV-K insertions (Additional file 1: Supplementary Methods and [18]). The code for the insertion call pipeline and simulation can be found at https://github.com/rsharris/suffynx

## Results

### Detecting retroviral integrations in LGL leukemia patient genomes

There is compelling evidence for retroviral involvement in LGL leukemia [19, 20]. Because a retrovirus must integrate into the host genome as part of its life cycle, we focused our methodology to detect retroviral insertions in the genomes of LGL cells, which are a clonal cell population, from LGL leukemia patients. Identifying a unique retrovirus integration site in a patient’s genome is complicated because reads derived from the novel retrovirus insertion will not map to a reference genome. Thus, we developed a two-step pipeline (Additional file 1: Supplementary Methods) that first detects insertion events in the patient genome and then determines if the insertion is of retroviral origin. Both steps of our method exploit specific mapping properties of long insert (8 kbp) mate pair data to identify retrovirus insertions (Fig. 1). The pipeline can detect retrovirus insertions of 5-8 kb if more than 55% of cells carry the insertion based on a simulation. Details of the pipeline and simulation are in Additional file 1: Supplementary Methods, Figure S1 and Additional file 2: Table S1.

We first applied this pipeline to the long insert mate pair WGS data of LGL leukemia patient S10, who had a PBMC count consisting of 80% clonal LGL cells. The first step of the insertion call pipeline is intentionally permissive to false positives because we implement a second step that utilizes the discordant mate pair data to detect retroviral sequences at each called insertion. Discordant mates identified at each called insertion site (represented in Track 4, Additional file 1: Figures S1 and S2) are assembled and queried against the NCBI database using BLAST+ [21, 22] (Additional file 1: Supplementary Methods). The majority of insertions were genome structural variants in the human population that are not in the hg19 reference genome but are represented in the NCBI nt database. A taxonomy ID and name search of the BLAST output using ‘retrovirus’ was applied to all contigs that were not identified as human. There were no detectable retrovirus matches in S10 sequence data using these criteria.

We further queried candidate insertions for key word ‘endogenous retrovirus’ (ERV), because there are polymorphic ERVs in the human population that are absent from hg19 [23, 24], many of which are represented in BAC clones; these would also appear as an insertion in our pipeline. Candidate human endogenous retrovirus (HERV) insertions were confirmed by two criteria: the contigs derived from unmapped mates mapped to a HERV in the NCBI nt database and the host regions flanking the HERV in the NCBI nt entry could be aligned to the reference human genome (hg19) in the interval defined by the anchoring mates near the called insertion. We detected four polymorphic HERV-K proviruses absent from hg19 in patient S10; these include two sites (chr1:73594980–73,595,948; chr10:27182399–27,183,380) containing a solo LTR in hg19, one site that had been previously reported as polymorphic (chr12:5727215–55,728,183) and one site that was recently reported to be polymorphic [23] (chr19:22414379–22,414,380), all of which we confirmed empirically. The pipeline was then used to analyze 10 additional LGL leukemia patient samples (S1-S9 and S11). None of the LGL patients had an unknown retrovirus sequence detectable in the DNA from PBMC but all patients had the polymorphic HERV-K proviruses at chr1:73594980–73,595,948 and chr10:27182399–27,183,380, while nine had the HERV-K at chr12:5727215–55,728,183 and five contained the chr19:22414379–22,414,380 HERV-K provirus.

The insertion call tool was designed to detect a retrovirus integration of 9 kbp or less in the clonally expanded LGL cells; a longer retrovirus or a retrovirus that integrated into non-leukemic cells would be below the level of sensitivity of our method. We took two additional approaches to search for a low frequency integration event in the sample. All unmapped reads that passed a quality filter were assigned taxonomy identification provided from SNAP [25] mapping (Additional file 1: Supplementary Methods). All reads with a ‘virus’ taxonomy classification were further scrutinized by BLAST search to determine if the best match was to a retrovirus. None were identified. We also mapped all long insert mate pair WGS reads to a full length HERV-K (GenBank accession number: JN675087) and assigned their mates to a position in hg19 to search for candidate low frequency de novo insertions of a HERV-K. Again, no unknown HERV-K proviral insertions were detected. Thus, we can confirm that there is not a clonal integration of an unknown exogenous or endogenous retrovirus in the LGL leukemia cells themselves and that we found no evidence of a novel retrovirus integration site in the genomes of non-leukemic cells that were represented in our WGS data.

This investigation was motivated by data showing that LGL patients have sero-recognition of HTLV proteins, although they are not infected with this virus [8, 19, 26]. Our detailed investigation of LGL genomes from 11 leukemia patients (S1-S11) failed to detect a novel retrovirus integration site in circulating LGL that could elicit this antibody response, but we did identify several HERV-Ks that are polymorphic in humans and absent from the human reference genome. Endogenous retroviruses have been implicated in several cancers including leukemia [27–32] and immune response to HERV proteins has been reported in both cancer and autoimmune diseases [29, 30, 33, 34]. Of the reported polymorphic HERV-K, 16 are close to full length [18, 23]. However, the genomic profile of polymorphic HERV-K proviruses in cancer patients compared to the population at large is at present unknown. We thus investigated the distribution of polymorphic HERV-K in LGL leukemia patients and normal populations represented in the KGP data [18, 35].

### Assessment of polymorphic HERV-K proviruses in the genomes of LGL leukemia patients

Our detection method to identify retroviral insertions used in this paper depended on long insert mate pair sequencing, which is not typically available for the large genomic databases needed to determine the prevalence of polymorphic HERV-K in global populations. We recently reported on a method that utilizes unique k-mers present in each published HERV-K provirus to estimate the proviral prevalence of polymorphic HERV-K proviruses in any individual from paired-end sequence data [18]. The output of the pipeline is the ratio (*n/T*) of k-mers from a query set (*n*) to the total number of unique k-mers (*T*) for each HERV-K proviral insertion. We applied this approach to 51 LGL leukemia patients, which included 11 (S1-S11) that were evaluated using our insertion call pipeline based on long-insert mate pair sequence data.

As previously noted [18], the distribution of polymorphic HERV-K proviruses varies considerably among KGP populations. LGL leukemia most frequently involves clonal proliferation of T cells and patients typically are of European descent although our sample of 51 LGL leukemia patients includes eight with NK cell leukemia and three individuals of non-European ancestry. Forty of the patients with T-LGL leukemia are of European origins, therefore we present the data both for all 51 LGL patients versus KGP and for the 40 T-LGL-EUR patients compared to European KGP data (EUR). Our analyses include 90 fixed and polymorphic HERV-K proviruses, omitting three on the Y chromosome, those recently reported to be expanding in centromeres [36, 37], and chr1:73594980–73,595,948 [18]. The provirus prevalence for both the entire LGL patient population (51 individuals) and the 40 T-LGL-EUR falls within the range of values for the five KGP populations for all HERV-K proviruses except those at chr1:75842771–75,849,143, chr12:58721242–58,730,698 and chr3:148281477–148,285,396, where prevalence in LGL patients is higher than any of the KGP populations (Table 1, see Additional file 3: Table S2 for the full data set); and chr19:21841536–21,841,542 and chr19:22414379–22,414,380, where LGL patients have a lower prevalence than any of the five KGP populations. The HERV-K provirus at chr12: 58721242–58,730,698 is noteworthy because 98% of T-LGL-EUR patients carry this HERV-K compared to 87% of EUR, which is the highest of the five KGP populations.

**Table 1:** Prevalence (proportion) of LGL patients and individuals from the five super-populations represented in the KGP data carrying a polymorphic HERV-K provirus

HERV-K (hg19 coordinate)	LGL	ELGL	KGP	AFR	AMR	EAS	EUR	SAS
chr1:75842771–75,849,143	70.6	70.0	42.9	26.8	56.5	6.0	68.9	66.8
chr3:148281477–148,285,396	49.0	52.5	41.9	38.9	42.6	45.0	46.5	37.4
chr12: 58721242–58,730,698	94.1	97.5	70.7	58.9	78.4	60.0	87.3	75.5
chr19: 21841536–21,841,542	5.9	7.5	27.0	39.2	11.9	32.2	10.7	32.4
chr19:22414379–22,414,380	51.0	47.5	67.8	89.2	60.8	56.9	55.8	67.2

*AFR* African, *AMR* Admixed American, *EAS* East Asian, *EUR* European, *SAS* South Asian, *LGL* All LGL patients in this study (51 total), *ELGL* T-LGL patients with European ancestry (T-LGL-EUR, 40 total)

### Validation of the polymorphic HERV-K proviral tool

We verified the estimates of HERV-K presence in LGL patients from our pipeline computationally and empirically. The four polymorphic HERV-K proviruses that are absent in hg19 were identified in our insertion call pipeline presented above using discordant long insert mate pair sequences for the 11 patients (S1-S11) analyzed. We also used the mate pair data to confirm the status of the remaining 16 polymorphic HERV-K that are represented in hg19; the results for HERV-K status based on our data mining tool and mate pairs are 100% concordant. In addition, we amplified both the preintegration site and a portion of HERV-K including the host flanking sequence for the four polymorphic HERV-K that were identified in the insertion call pipeline in 48 of the LGL patients (LGL) and 48 individuals with no diagnosed diseases (European, African-American and Hispanic origin; hereafter referred to as “normal”). The results from the PCR assay for chr19:22414379–22,414,380 (normal 56%, LGL patient 50%) and chr12:55727215–55,728,183 (normal 82%, LGL patients 71%) agree with our computational analysis (Additional file 3: Table S2). The chr10:27182399–27,183,380 HERV-K was amplified in all LGL patients and normal individuals, which is consistent with the high prevalence (99%) found in our data mining method. The HERV-K provirus at chr1:73594980–73,595,948 was also present in all patients and normals by PCR but we have no data on this virus from our KGP analysis because the build of the reference genome (GRCh37) used to map KGP reads included hs37d5, a concatenated decoy sequence which contains this virus, while our approach used coordinates of hg19. Hence the reads needed to identify the HERV-K at chr1:73594980–73,595,948 were not extracted in the data mining step [18].

### Comparison of HERV-K proviral distribution in LGL leukemia patients and KGP

**Fig. 2:**
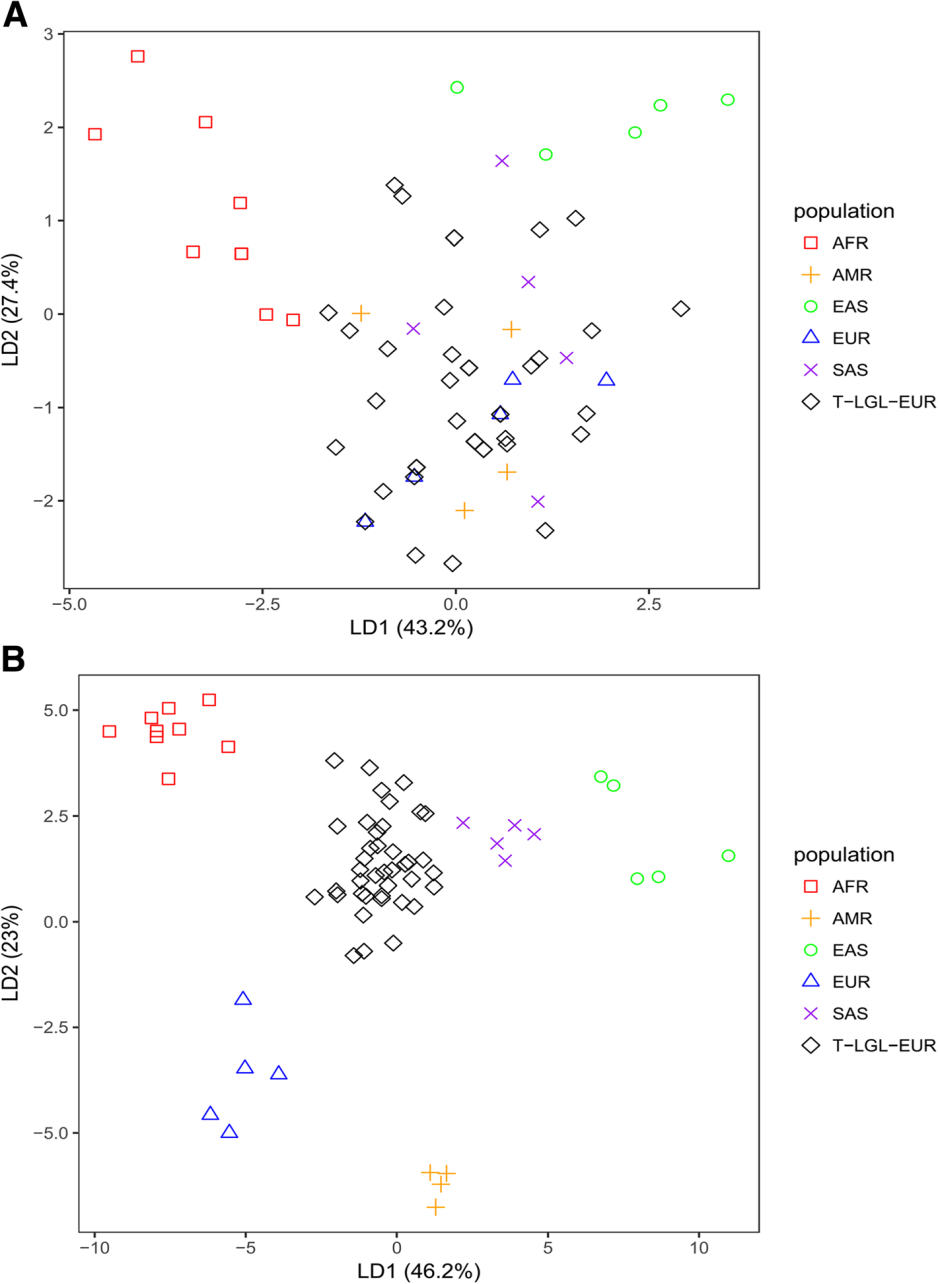
Linear discriminant analysis based on HERV-K status of T-LGL-EUR patients and KGP super populations. Linear discriminant analysis (LDA) was conducted on data generated by a comprehensive analysis of polymorphic HERV-Ks in an individual genome [18]. **a** Data is based on three HERV-K states of ‘absence,’ ‘solo LTR’, ‘provirus’ or **b.** The *n/T* ratio of each known HERV-K provirus for T-LGL leukemia patients of European ancestry and the 28 individuals from KGP super populations with high coverage data. The ratio indicates the proportion of k-mers derived from a person’s WGS dataset (*n*) that are 100% match to a set of unique k-mers (*T*) characterizing each HERV-K provirus. The improved resolution of T-LGL-EUR patients from other individuals using *n/T* likely reflects that alleles of HERV-K contribute to population differentiation. The symbols and colors for each KGP populations and T-LGL-EUR leukemia patients are indicated in the key on the right

We performed a linear discriminant analysis (LDA) to determine if the signal in the HERV-K prevalence data was sufficient to distinguish the T-LGL-EUR patient population from EUR KGP individuals. For this analysis we used only the 28 individuals from the KGP data sets with high coverage (~30x sequencing depth) data, after confirming that none were outliers in their population clusters of all KGP data (~5x sequencing depth) [18]. Based on the data reduced to the states ‘absence, solo LTR, provirus’ of each HERV-K insertion, T-LGL-EUR patients separate from EAS and AFR but were admixed with EUR, AMR and SAS (Fig. 2a). We previously showed that using the *n/T* ratio provided better resolution of the KGP populations [18], presumably because it captures allelic differences in both fixed and polymorphic HERV-K. Based on *n/T*, T-LGL-EUR patients are well separated from all KGP populations (Fig. 2b). These data indicate that both the polymorphic HERV-K and specific allelic forms of each HERV-K provirus define the T-LGL patient population.

An *n/T* ratio of 1 indicates that the reference allele (typically from hg19) is present. We suggested that *n/T* less than 1 indicates that the query HERV-K at a given locus is an allele that is not represented in the database [18]. Using the unmapped mates from those reads flanking a HERV-K locus, we reconstructed the sequence of a polymorphic HERV-K provirus at chr3:112743479–112,752,282, which is presented here because there is considerable variation in *n/T* in both LGL patients (Additional file 1: Figure S4A) and KGP [18]. For individuals with *n/T* = 1, all unique k-mers for the reference alleles are found in the patient data. However, LGL patients with *n/T* < 1 have five substitutions in this HERV-K, one common to the 11 patients (S1-S11) with long insert mate pair data and four sites that were variably present among these individuals (Additional file 1: Figure S4B), which accounts for their lower values of *n/T*. These data demonstrate that *n/T* does reflect allelic differences at a HERV-K locus and indicate why the *n/T* ratio contains more information than the presence, absence data to distinguish populations.

### Determining the total burden of polymorphic HERV-K in LGL leukemia patient genomes

**Fig. 3:**
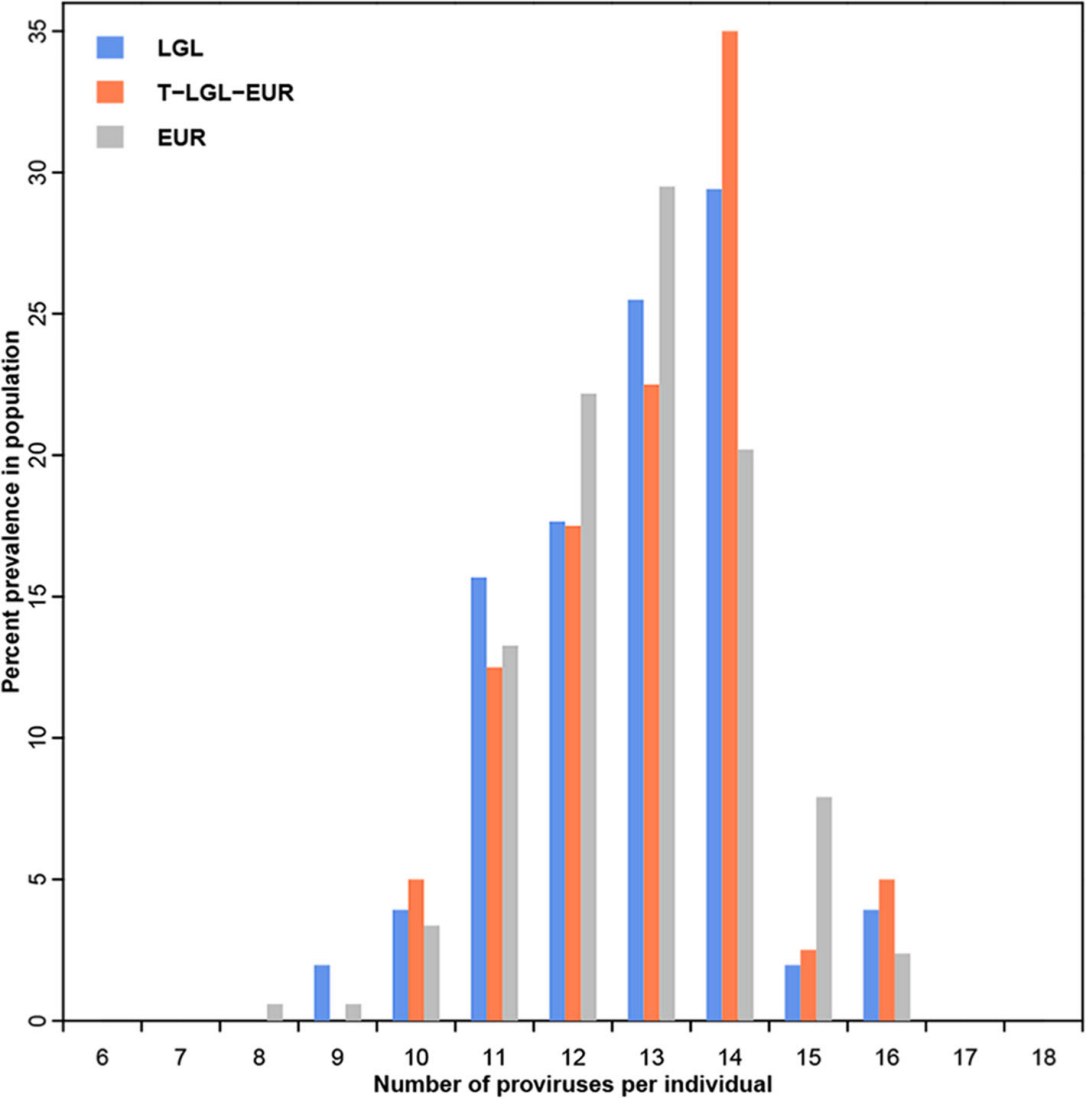
Histogram of the number of polymorphic HERV-K proviruses identified in LGL leukemia patients compared to individuals of European origin from KGP. Data are shown for 51 LGL patients (blue) and for the subset of 40 patients with T-LGL leukemia of European ancestry (T-LGL-EUR, orange). Data for the 505 EUR individuals (gray) from the KGP data is from Li et al. [18]

Genomic structural variations are often noted in cancer cells of diverse origins [38, 39]. Because HERV-Ks are polymorphic in the genome some individuals could have a higher burden of these repetitive elements than others. We considered that an increased number of polymorphic HERV-K proviruses could contribute to the sustained clonal proliferation that characterizes LGL leukemia by increasing structural variation [40–42]. In the KGP datasets, no individual had fewer than 7 or more than 18 of the 20 polymorphic HERV-K proviruses evaluated and ~ 50% of all individuals from each of the KGP populations have 12 or 13 polymorphic provirus insertions except for EAS, where 52% of the sampled individuals have 9–11 polymorphic integration sites [18]. The genomes of T-LGL-EUR patients contain between 10 and 16 HERV-K proviruses (Fig. 3) at proportions that are significantly different than those found in EUR individuals (Kolmogorov-Smirnov test, *p* = 0.0087; Additional file 3: Table S2). Notably, 35% of T-LGL-EUR individuals carry 14 proviruses while the carriage rate for this number of HERV-K proviruses among the five KGP populations is 2–22%. Hence, the LGL leukemia patient genomes sampled in this analysis contain a higher burden of polymorphic HERV-K than is seen in the general population.

### Estimating co-occurrence of polymorphic HERV-K proviruses in LGL leukemia genomes

**Fig. 4:**
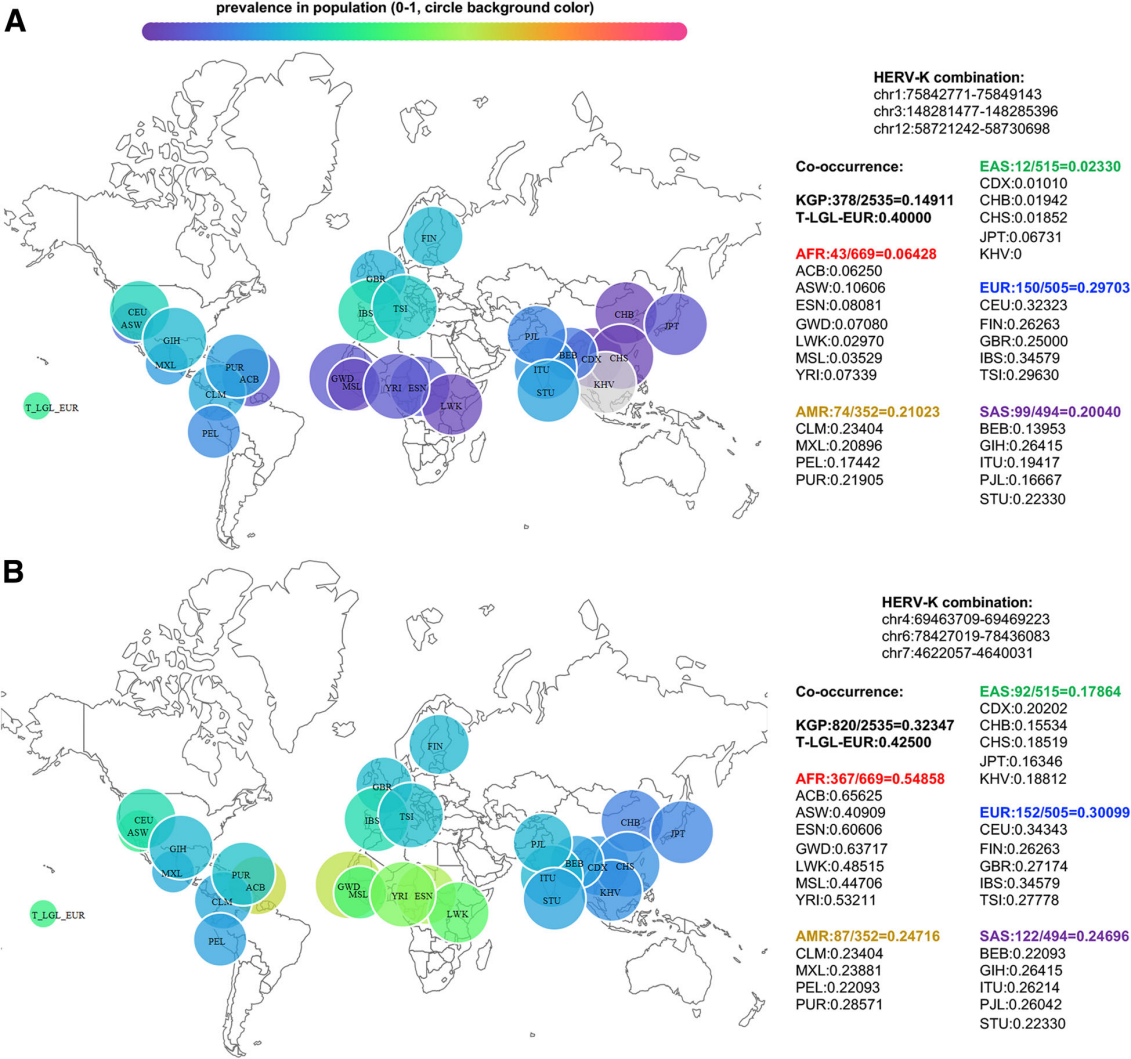
The prevalence of combinations of polymorphic HERV-K provirus in KGP populations and T-LGL-EUR leukemia patients. The combinations of polymorphic HERV-K provirus evaluated are indicated at the top right of each panel. **a** The prevalence of three polymorphic HERV-K proviruses that include chr12: 58721242–58,730,698 in KGP individuals and T-LGL-EUR patients. **b** The prevalence of three polymorphic HERV-K, excluding chr12: 58721242–58,730,698, in KGP individuals and T-LGL-EUR leukemia patients. Coordinates are referenced to hg19. Bubble size is proportional to the number of individuals in the population and color gradient represents prevalence from 0 to 100%. Absolute values are given in the text on the right for each population. KGP population abbreviations are given in Table 1 and additional information can be found at (http://www.internationalgenome.org/category/population/)

Because LGL leukemia patients, and particularly T-LGL leukemia patients, have more HERV-K proviruses in their genomes, we reasoned that co-occurrence of the polymorphic HERV-Ks could also vary from EUR or other global populations. This is the case for several HERV-K combinations that include chr12: 58721242–58,730,698, which is present in 98% of T-LGL leukemia patients (Fig. 4a). However, there are other combinations of HERV-K, with or without the provirus at chr12: 58721242–58,730,698, that are higher in LGL patients than in EUR but similar to AFR and EAS populations (Fig. 4b). Co-occurrence of polymorphic HERV-K provirus should be considered when investigating a role of HERV-K in the pathogenesis of a specific disease because even defective retroviruses are capable of both recombination and complementation to generate a progeny with a novel phenotype [43–46].

## Discussion

Our goal was to determine if the serological reactivity to retroviral antigens reported in LGL leukemia patients was indicative of a novel retrovirus integration in LGL genomes, which could be responsible for the clonal expansion of LGL that characterize this leukemia. Insertional mutagenesis is in part a consequence of where the retrovirus integrates in the genome. It is a common mechanism of retroviral oncogenesis because proximity to a host gene can result in both altered regulation of the gene and expression of the retrovirus [47, 48]. If retrovirus integration preceded the clonal expansion of LGL, the insertion site should be present in the genome of leukemic cells from LGL leukemia patients at a high enough frequency for us to detect. Our detailed analysis of insertion sites in LGL leukemia patient genomes demonstrates that there is no new retroviral integration site in the clonally expanded cells. This is an important finding because it directs research on retroviral involvement in LGL leukemia towards the many other mechanisms by which retrovirus can cause cancer. Of particular interest, our computational efforts revealed several polymorphic HERV-Ks in LGL leukemia patient genomes that are absent in the human reference genome. The role of HERV-Ks in human disease is an exciting and active research area as tools become available to study theses repetitive genome elements, which are polymorphic in human genomes. Hence, we applied our recently developed tools [18] to investigate the genome wide distribution of HERV-K in LGL leukemia patients compared with unaffected individuals represented in the KGP data.

The most notable difference between LGL leukemia patients and individuals represented in KGP data is in the increased number of polymorphic HERV-K proviruses that they carry. The difference is more pronounced when restricting the comparisons to the 40 individuals with T-LGL leukemia who are of European ancestry (T-LGL-EUR). This likely reflects the fact that EAS populations represented in KGP have a significantly lower overall burden of polymorphic HERV-K and there are two LGL leukemia patients of East Asian descent in our test cohort. LGL leukemia patients also have an elevated prevalence of chr12:58721242–58,730,698 and several combinations of polymorphic HERV-K are found more frequently in LGL leukemia patients than in EUR individuals, although not all involve the chr12:58721242–58,730,698 HERV-K. HERVs can contribute to structural variation in the genome by non-homologous recombination and gene conversion mechanisms [49–53]. Because our data suggest that LGL leukemia patients carry more total HERV-K proviruses than non-leukemic individuals, detailed analyses of structural variants in proximity to HERV-K proviral loci is warranted.

Our previous analysis of the KGP data also suggested that there were alleles of HERV-K proviruses not found in the NCBI databases; these are represented by an *n/T* ratio of less than 1 [18]. We reconstructed the provirus sequences at HERV-K loci using patient long insert mate pair WGS data to confirm that our analysis tool does report allelic differences in HERV-K that are not found in any of the reference HERV-K that localize to that site. We call these unknown alleles because we require 100% match of query k-mers from patient WGS to the set of k-mers, *T*, which represent k-mers unique to all alleles present in public databases of a HERV-K at a specific locus. If a k-mer derived from a patient contains a sequence polymorphism at any position of the unique reference k-mer set *T*, it will be excluded in the k-mer count, effectively decreasing *n/T* to less than 1. It is notable that there is substantial variation in *n/T* for both fixed and polymorphic HERV-K and these differences, not presence or absence of a HERV-K, distinguish the KGP super-populations [18]. Although population-specific alleles have been reported [54], our data highlight more extensive sequence variation among HERV-Ks than has previously been recognized and suggest that both proviral sequence and site occupancy should be considered when assessing the potential role of HERV-K in disease. This is an important consideration because using a consensus sequence or specific reference sequence might not reflect the sequence of a HERV-K provirus in a patient population. While our analyses to date reflect the genomic composition of proviral HERV-K and we have no data on proviral expression, such studies are clearly warranted to determine if HERV-K could contribute to LGL leukemia pathogenesis by mechanisms involving viral RNA and protein; reviewed in [55].

The data we present do not rule out the contribution of an infectious retrovirus to LGL leukemia pathogenesis. The insertion call pipeline detects a retrovirus insertion present in the genomes of more than 55% of the sampled cells; therefore, a clonal integration of a new retrovirus in the LGL leukemia cells, which comprise greater than 60% of the peripheral mononuclear cell population or a new germline insertion of HERV-K provirus would be identified. However, a retrovirus infecting another tissue could express viral antigen that is responsible for stimulating the observed antibody response. Although no sero-reactivity to HERV-K Gag or Pol antigens was found in LGL leukemia patients [56] it is possible that there is an antibody response to other epitopes of an aberrantly expressed HERV-K [27]. Given the well-established ability of infectious retroviruses to activate and recombine with ERVs [35, 44, 57–61], an additional and intriguing consideration is that LGL from leukemia patients respond to an antigen from a chimeric, replication-incompetent retrovirus. Further immunological analyses to understand the nature of the antigens that can either induce an anti-retroviral response or sustain proliferation of LGL, or both, will provide insight on the role of retroviruses in the pathogenesis of LGL leukemia.

## Conclusions

Our results indicate that LGL leukemia patients have a genomic profile of polymorphic HERV-K provirus that is different than populations at large. Thus, a thorough analysis of HERV-K loci may reveal if they induce structural or epigenetic variation in the host genome that could contribute to the pathogenesis of this leukemia. Future studies should also detail HERV-K expression profiles in activated T cells and LGL to determine if retroviral seroresponse is to HERV-K or if HERV-K expression influences pathogenesis in LGL leukemia patients. Because a considerable number of LGL leukemia patients also have an autoimmune disease, further comprehensive investigation of the roles of endogenous or exogenous retroviruses in LGL leukemia and autoimmune disease is indicated.

## Additional files

Additional file 1: Supplementary Methods and Data. **Figures S1-S4.** are embedded. (DOCX 3556 kb)

Additional file 2: **Table S1.** Results of 10 simulated runs to determine how the proportion of infected cells and the length of the retrovirus affects detection by the insertion pipeline. (XLSX 9 kb)

Additional file 3: **Table S2.** The output of the pipeline to investigate HERV-K proviruses and prevalence of polymorphic HERV-K proviruses in 51 LGL leukemia patients. (XLSX 68 kb)

## Data Availability

The complete WGS datasets analyzed during the current study are not publicly available because a full analysis of the genome sequence for other purposes is ongoing. However, the bam files used for analysis of HERV-K provirus occupancy will be made publically available when the manuscript is accepted for publication. The long insert mate pair data from 11 patients are available from the corresponding author on reasonable request. Code for the insertion call pipeline and the simulation along with documentation is at: https://github.com/rsharris/suffynx

## Abbreviations

ERV: Endogenous retrovirus
HERV: Human endogenous retrovirus
HERV-K: Human endogenous retrovirus type K
HTLV: Human T-lymphotropic virus
KGP: 1000 genomes project
LGL: Large granular lymphocyte
WGS: Whole genome sequencing
